# The Predictive Role of the Gleason Score in Determining Prognosis to Systematic Treatment in Metastatic Castration-Sensitive Prostate Cancer: A Systematic Review and Network Meta-Analysis

**DOI:** 10.3390/jcm14041326

**Published:** 2025-02-17

**Authors:** Yao-Cheng Wu, Shiow-Ing Wang, Li-Yu Lu, Min-You Wu, Pei-Lin Wu, Tzuo-Yi Hsieh, Wen-Wei Sung

**Affiliations:** 1 School of Medicine, Chung Shan Medical University, Taichung 402306, Taiwan; s0801086@gm.csmu.edu.tw (Y.-C.W.); s0801048@gm.csmu.edu.tw (L.-Y.L.); s0701130@gm.csmu.edu.tw (M.-Y.W.); s0801132@gm.csmu.edu.tw (P.-L.W.); 2 Center for Health Data Science, Department of Medical Research, Chung Shan Medical University Hospital, Taichung 402306, Taiwan; cshe1301@csh.org.tw; 3 Department of Health Policy and Management, College of Health Care and Management, Chung Shan Medical University, Taichung 402306, Taiwan; 4 Institute of Medicine, Chung Shan Medical University, Taichung 402306, Taiwan; 5 Department of Urology, Chung Shan Medical University Hospital, Taichung 402306, Taiwan

**Keywords:** prostate malignancy, survival, androgen receptor signaling inhibitors, androgen deprivation therapy, rezvilutamide, darolutamide

## Abstract

**Background**: Gleason scores of 8 or higher indicate a poorer prognosis in metastatic castration-sensitive prostate cancer (mCSPC). This study aims to perform a systematic review and network meta-analysis (NMA) to compare overall survival (OS) and progression-free survival (PFS) among combination therapies with androgen receptor signaling inhibitors (ARSIs) in mCSPC patients, stratified by Gleason score ≥8 and <8. **Methods**: A literature search was conducted across PubMed, Embase, and Web of Science, using a PRISMA-guided systematic search strategy, covering January 2013 to June 2024. **Results**: Twelve studies including 12,652 patients were included in the NMAs. In the overall population, most ARSI combination therapies improved survival outcomes, except for orteronel + androgen deprivation therapy (ADT). In the Gleason score ≥8 subgroup, all ARSI combination therapies improved OS, with rezvilutamide showing the highest probability of being the best treatment for OS (HR 0.48, 95% CI 0.31–0.76, P-scores 0.88). In the Gleason score <8 subgroup, only darolutamide + docetaxel + ADT (HR 0.49, 95% CI 0.29–0.81) and apalutamide + ADT (HR 0.67, 95% CI 0.46–0.98) improved OS. **Conclusions**: ARSI combination therapy is effective for mCSPC patients with Gleason score ≥8, but further investigation is needed to confirm its efficacy in patients with Gleason score <8.

## 1. Introduction

Prostate cancer, one of the major malignancies affecting men, is the second leading cause of cancer death among men in the United States [1]. The incidence rate of prostate cancer experienced a 3% annual increase from 2014 to 2019, with about half of these cases classified as advanced. The incidence and mortality of prostate cancer worldwide are associated with advancing age, with an average age of 66 years at the point of diagnosis [2]. Clinically, prostate cancer is typically identified through methods such as digital rectal examination, prostate-specific antigen (PSA) blood test, transrectal ultrasound (TRUS)-guided biopsy, or magnetic resonance imaging (MRI) [3]. In the USA and Europe, the implementation of early detection strategies based on PSA testing has led to a rise in identifying indolent disease and a reduction in the diagnosis of metastatic prostate cancer [4].

While most prostate cancer patients initially present with localized cancer [1], a proportion of them will eventually experience recurrence after treatment [5]. Since the growth of prostate cancer is driven by androgens, especially testosterone and dihydrotestosterone, androgen deprivation therapy (ADT) has been the primary approach for managing metastatic prostate cancer, as demonstrated by Huggins and Hodges through castration therapy in the 1940s [6]. For metastatic prostate cancer, ADT has been the standard of care (SOC). The majority of recurrent cases initially respond to ADT, known as metastatic castration-sensitive prostate cancer (mCSPC). However, as resistance develops over time, mCSPC transforms into metastatic castration-resistant prostate cancer (mCRPC), characterized by disease progression and a poor prognosis despite castrate levels of serum testosterone [7]. For mCSPC, therapeutic decisions and the design of clinical trials are often guided by an evaluation of risk. High-risk prognostic factors for mCSPC include a Gleason score of ≥8, a prostate-specific antigen (PSA) level of >20 ng/mL, or a clinical stage of ≥T3, indicating a poorer prognosis [8].

Reducing testosterone levels to below “castrate” levels (testosterone < 50 ng/dL), whether through bilateral orchiectomy or medical castration with luteinizing hormone-releasing hormone agonists or antagonists, has long been a standard treatment for mCSPC. However, with the introduction of docetaxel and ADT combination therapy [9,10], which has improved overall survival (OS) compared to ADT monotherapy, the treatment strategies have changed. Additionally, increasing evidence has shown that doublet therapies that combine ADT with either the chemotherapy agent docetaxel [9,10,11,12,13,14] or an androgen receptor signaling inhibitor (ARSI), such as abiraterone acetate [15,16,17,18,19], apalutamide [20,21], enzalutamide [22,23,24,25], rezvilutamide [26], or orteronel [27], result in better survival rates than ADT alone.

Recently, the results of the ARASENS (darolutamide + docetaxel + ADT) [28] and PEACE-1 (abiraterone acetate + docetaxel + ADT) [29] trials showed that ARSI-based triplet therapy provided better OS outcomes than standard ADT.

The Gleason score is a grading system for prostate cancer based on its microscopic appearance from a prostate biopsy. A Gleason score of 8 or greater is considered high grade with a poorer prognosis compared to low grade, which is characterized by a Gleason score of less than 8 [30]. However, the impact of ARSI in different Gleason score subgroups has not yet been thoroughly researched. In this systematic review and network meta-analysis (NMA), we systematically evaluated the OS and progression-free survival (PFS) in mCSPC patients treated with the chemotherapy agent docetaxel or ARSI agents. Subsequently, we compare the OS between subgroups of mCSPC patients with or without a Gleason score of ≥8.

## 2. Methods

### 2.1. Study Protocol

This systematic review and meta-analysis followed the Preferred Reporting Items for Systematic Reviews and Meta-Analyses (PRISMA) [31]. We registered the study protocol in the International Prospective Register of Systematic Reviews database (PROSPERO: CRD42024574367).

### 2.2. Literature Search

Four authors (Y.C.W., M.Y.W., L.Y.L., P.L.W.) independently searched three electronic databases, namely, PubMed, Embase, and Web of Science, for publications dating from January 2013 to June 2024. Any inconsistencies in the search results were resolved through consensus. Our search focused on the following keywords: “enzalutamide”, “darolutamide”, “apalutamide”, “abiraterone acetate”, “rezvilutamide”, “orteronel”, “docetaxel”, “anti-androgens”, “antiandrogens”, “hormonal therapy”, “metastatic”, “prostate cancer”, and “randomized”. The detailed search strategy is presented in Supplementary Table S1. Additionally, we reviewed the references of related articles for any additional relevant studies.

### 2.3. Study Selection

After removing duplicates, two investigators (Y.C.W. and P.L.W.) examined the study titles and abstracts. Subsequently, the full texts of the relevant studies were reviewed and considered eligible for inclusion if they satisfied the following inclusion criteria: 1. patients diagnosed with metastatic castration-sensitive prostate cancer (mCSPC), aged 18 years or above; 2. the treatment group was treated with anti-androgen receptor signaling inhibitors (ARSIs) or docetaxel; 3. the control group received standard treatment (androgen deprivation therapy ± first-generation nonsteroidal anti-androgens or docetaxel); 4. the study outcome focused on overall survival (OS); 5. randomized control trial; 6. written in English. Studies were excluded based on the following exclusion criteria: 1. case reports, case series, cohort studies, case–control studies, nonrandomized clinical trials, and self-control trials; 2. studies without available full text; 3. conference posters.

### 2.4. Data Extraction

Two authors (M.Y.W. and L.Y.L.) extracted the following data independently: study design and first author, year of publication, overall population, patient numbers for Gleason scores of ≥8 and <8, mean age, median follow-up time, and hazard ratio for all groups. The Gleason system is based on the arrangement of glands in prostate tumor tissue specimens. Glandular arrangements that are closest to normal cells are classified as grade 1, while those that are most disordered are classified as grade 5. The Gleason score is the sum of the grades of the largest and the second-largest areas.

### 2.5. Quality Assessment

Two authors (M.Y.W. and L.Y.L.) performed quality assessments independently using version 2 of the Cochrane risk-of-bias tool for randomized trials (RoB 2) [32]. Disagreements were discussed with another author, and the decision was made by consensus. The RoB 2 scores were assessed using five indicators: randomization process, deviations from intended interventions, missing outcome data, measurement of the outcome, and selection of the reported result. All indicators that qualified were considered high quality.

### 2.6. Statistical Analysis

The primary analysis evaluated OS using the Mantel–Haenszel random-effects model to compare mCRPC systemic treatments against the reference treatment, ADT. Subgroup analyses were conducted based on patients’ Gleason scores (≥8 or <8) to evaluate and compare OS within each group. The hazard ratio (HR) and corresponding 95% confidence interval (CI) for OS were calculated and then pooled in a meta-analysis using statistical software R 4.3.1 (R Foundation for Statistical Computing, Vienna, Austria). Statistical significance was defined at *p* < 0.05. The odds ratios (ORs) for all potential regimen comparison outcomes were displayed using league tables. The results of the NMA are presented graphically using forest plots and sorted by P-scores, compared to ADT. Higher P-scores indicate a greater treatment efficacy. We used I^2^ statistics to evaluate heterogeneity. I^2^ > 50% indicates high heterogeneity. Trials exhibiting high heterogeneity were excluded, maintaining I^2^ values below 50%. Begg’s funnel and Egger’s test were used to estimate publication bias.

## 3. Results

### 3.1. Characteristics of Included Studies

**Figure 1:**
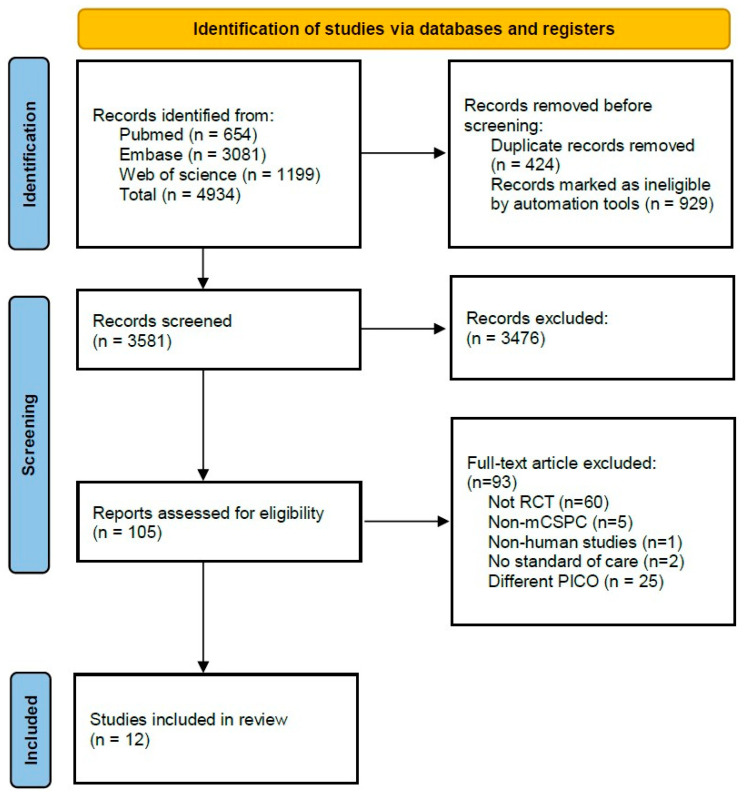
The PRISMA flow chart detailing the article selection process. RCT, randomized controlled trial.

We first identified 4934 publications. After duplicates were eliminated, 3581 records remained for screening of titles, abstracts, and full texts (Figure 1). Finally, we identified 12 RCTs that met the inclusion criteria of our systematic review and NMA according to our selection criteria [10,11,13,15,16,20,22,24,26,27,28,29]. The extracted data from each included study are outlined in Table 1. These studies, published between 2013 and 2022, involved a total of 12,652 patients. The most investigated doublet treatments were docetaxel (three trials) [10,11,13], abiraterone acetate (two trials) [15,16], and enzalutamide (two trials) [22,24]. Additionally, one trial each studied ADT combined with apalutamide [20], rezvilutamide [26], or orteronel [27]. For triplet therapies, two trials were investigated (darolutamide + docetaxel + ADT and abiraterone acetate + docetaxel + ADT) [28,29]. In seven trials, the comparator was either a placebo or no treatment, while in three trials, it was standard nonsteroidal anti-androgen (NSAA) therapy (including bicalutamide, nilutamide, or flutamide). Network graphs of the trial comparison are shown in Supplementary Figures S1–S4.

### 3.2. Risk of Bias Assessment

**Figure 2:**
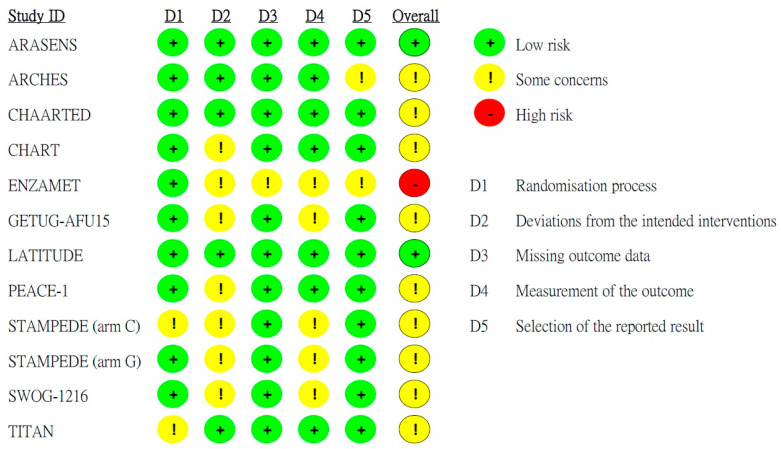
Quality assessment for risk of bias of each included RCTs. The quality assessment was performed using version 2 of the Cochrane risk-of-bias tool (RoB 2).

The risk of bias in the included studies is summarized in Figure 2. One study was judged to be at high risk of bias due to concerns across multiple domains. Nine studies were rated as unclear risk of bias because one or more criteria were evaluated as unclear. Two studies were categorized as having a low risk of bias, as each domain received an assessment indicating low risk. Overall, the quality of the studies was assessed as medium. The ENZAMET study conducted by Davis et al. [24] was classified as high risk due to inadequate description of randomization, lack of concealment in treatment allocation, and insufficient reporting on the handling of missing data.

### 3.3. Quality of the Evidence

To explore the heterogeneity among studies in mCSPC, we performed a homogeneity analysis (Supplementary Figures S5–S8). Statistical heterogeneity was assessed using the I^2^ statistic, where an I^2^ value exceeding 50% indicated substantial heterogeneity. We pooled data from multiple trials, excluding those with high levels of heterogeneity.

### 3.4. Efficacy Outcomes

**Figure 3:**
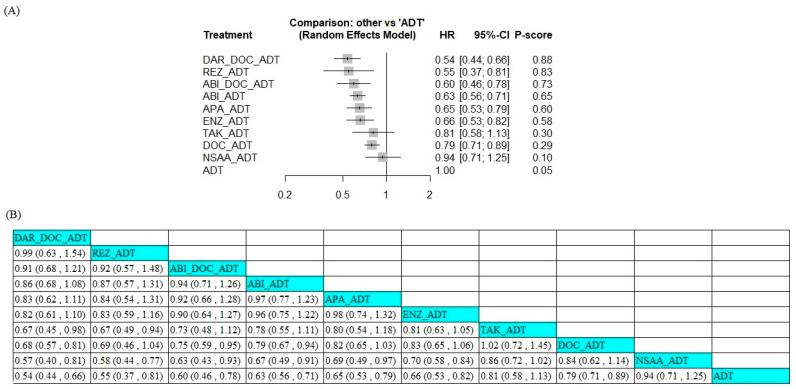
Forest plot of hazard ratios (**A**) and league table (**B**) results for overall survival across different therapeutic regimens. ABI, abiraterone acetate; ADT, androgen deprivation therapy; APA, apalutamide; DAR, darolutamide; DOC, docetaxel; ENZ, enzalutamide; NSAA, standard nonsteroidal anti-androgen (bicalutamide, nilutamide, or flutamide); REZ, rezvilutamide; TAK, orteronel.

Twelve trials were included in the OS analysis. Ten studies involving doublet therapy were pooled, including treatments with ADT plus either the chemotherapy agent docetaxel or ARSI agents such as abiraterone acetate, apalutamide, enzalutamide, darolutamide, rezvilutamide, and orteronel (ARCHES [22,23], CHAARTED [9,11], CHART [26], ENZAMET [24,25], GETUG-AFU15 [13,14], LATITUDE [15,17], STAMPEDE arm C [10], STAMPEDE arm G [19], SWOG-1216 [27], and TITAN trials [20,21]). Additionally, two trials involving triplet therapy were pooled, namely, abiraterone acetate + docetaxel + ADT and darolutamide + docetaxel + ADT (PEACE-1 and ARASENS). Most ARSI doublet or triplet therapies improved survival outcomes compared to ADT alone, except orteronel + ADT (Figure 3A). Darolutamide + docetaxel + ADT had the lowest hazard ratio (HR) compared to ADT alone (HR 0.54, 95% CI 0.44–0.66). According to P-score ranking, this triplet therapy also had the highest probability of being the best treatment regarding OS, with a P-score of 0.88, followed by rezvilutamide (0.83), abiraterone acetate + docetaxel (0.73), abiraterone acetate (0.65), apalutamide (0.60), enzalutamide (0.58), orteronel (0.30), docetaxel (0.29), and NSAA (0.10). The league table is shown in Figure 3B.

In the overall PFS comparison, the analysis was derived from 11 trials reporting on PFS. The ARASENS trial was excluded due to a lack of PFS data. ARASENS, ARCHES, CHART, ENZAMET, and SWOG-1216 were excluded due to high heterogeneity. All ARSI combination treatments improved PFS more than ADT alone (Supplementary Figure S9). Triplet therapy with abiraterone acetate + docetaxel + ADT was the most effective therapeutic regimen compared with ADT alone (HR 0.33, 95% CI 0.23–0.49, P-score 0.99).

### 3.5. Efficacy in Patients with Gleason Score ≥ 8

**Figure 4:**
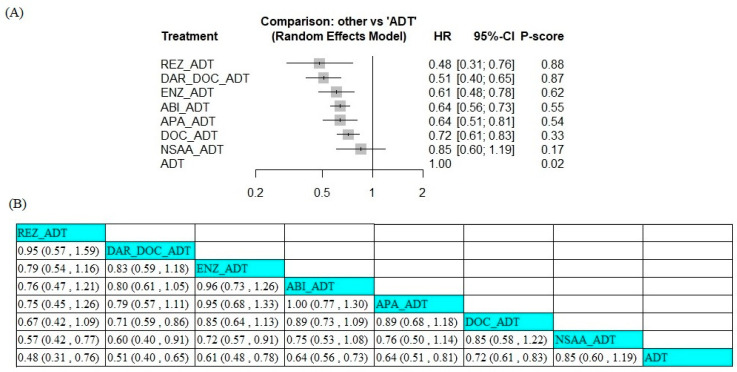
Forest plot of hazard ratios (**A**) and league table (**B**) results for overall survival across different therapeutic regimens in patients with a Gleason score of ≥8. ABI, abiraterone acetate; ADT, androgen deprivation therapy; APA, apalutamide; DAR, darolutamide; DOC, docetaxel; ENZ, enzalutamide; NSAA, standard nonsteroidal anti-androgen (bicalutamide, nilutamide, or flutamide); REZ, rezvilutamide.

We conducted an NMA that comprised ten trials for analyzing the survival outcome in mCSPC patients with a Gleason score of ≥8. In this subgroup analysis, one trial involving triplet therapy, six trials involving ARSI + ADT, and two trials involving docetaxel + ADT were pooled, except GETUG-AFU15 due to high heterogeneity. The SWOG-1216 and PEACE-1 trials were excluded due to not reporting the Gleason score data. Compared with ADT alone, all systemic therapies including ARSI or docetaxel improved OS in Gleason score ≥8 mCSPC patients (Figure 4A), while NSAA did not. The P-score ranking showed that rezvilutamide had the highest probability of being the best treatment regarding OS (HR 0.48, 95% CI 0.31–0.76), with a P-score of 0.88, followed by darolutamide + docetaxel (0.87), enzalutamide (0.62), abiraterone acetate (0.55), apalutamide (0.54), docetaxel (0.33), and NSAA (0.17). The pairwise analyses are presented in Figure 4B.

### 3.6. Efficacy in Patients with Gleason Score < 8

**Figure 5:**
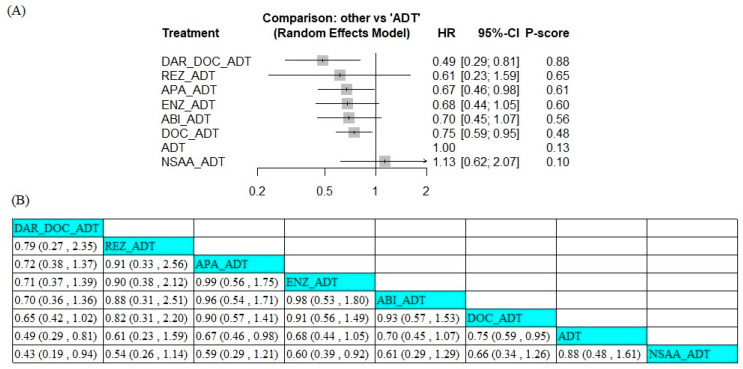
Forest plot of hazard ratios (**A**) and league table (**B**) results for overall survival across different therapeutic regimens in patients with a Gleason score of <8. ABI, abiraterone acetate; ADT, androgen deprivation therapy; APA, apalutamide; DAR, darolutamide; DOC, docetaxel; ENZ, enzalutamide; NSAA, standard nonsteroidal anti-androgen (bicalutamide, nilutamide, or flutamide); REZ, rezvilutamide.

We conducted an NMA that comprised ten trials for analyzing the survival outcome in mCSPC patients with a Gleason score of <8. In this subgroup analysis, one trial involving triplet therapy, six trials involving ARSI + ADT, and three trials involving docetaxel + ADT were pooled. In the Gleason score ≥8 subgroup, SWOG-1216 and PEACE-1 trials were excluded due to not reporting the Gleason score data. Among ARSI combination therapies, darolutamide + docetaxel + ADT (HR 0.49, 95% CI 0.29–0.81) and apalutamide +ADT (HR 0.67, 95% CI 0.46–0.98) improved OS in Gleason score <8 mCSPC patients (Figure 5A). The P-score ranking showed that darolutamide + docetaxel + ADT had the highest probability of being the best treatment regarding OS, with a P-score of 0.88, followed by rezvilutamide (0.65), apalutamide (0.61), enzalutamide (0.60), abiraterone acetate (0.56), docetaxel (0.48), and NSAA (0.10). The pairwise analyses are presented in Figure 5B.

### 3.7. Publication Bias

To identify publication bias, we created funnel plots (Supplementary Figures S10–S13). In these plots, the vertical axis represents the standard error, placing larger studies at the top and smaller ones at the bottom. The horizontal axis shows the power and effect sizes of the included studies. The funnel plot revealed no asymmetry. Subsequently, Egger’s test was performed to conduct a numerical assessment of only two of the funnel plots, as a minimum of ten studies was required. Both *p*-values are greater than 0.05, indicating no evidence of publication bias.

## 4. Discussion

This study provides the latest evidence on the efficacy of combined androgen receptor signaling inhibitor (ARSI) and/or docetaxel with androgen deprivation therapy (ADT) for treating metastatic castration-sensitive prostate cancer (mCSPC) patients with a Gleason score of ≥8 or <8, a critical issue not yet addressed in clinical trials. The Gleason score is important in managing metastatic prostate cancer, influencing disease prognosis and treatment decisions. Knowing the Gleason score before treatment in mCSPC patients helps clinical decision making. Through a comprehensive review of eligible randomized controlled trials (RCTs), we report that, with the exception of orteronel + ADT, most systemic therapies including ARSIs improved survival outcomes compared to ADT alone. Specifically, all therapies with ARSI + ADT or ARSI + docetaxel + ADT improved overall survival (OS) in the Gleason score ≥ 8 subgroup, with rezvilutamide showing the highest probability of being the best treatment for OS (HR 0.48, 95% CI 0.31–0.76, P-score 0.88). However, in the Gleason score <8 subgroup, only darolutamide + docetaxel + ADT (HR 0.49, 95% CI 0.29–0.81) and apalutamide + ADT (HR 0.67, 95% CI 0.46–0.98) significantly improved OS.

ADT is the cornerstone of mCSPC treatment, reducing testosterone levels to below castrate levels to inhibit prostate cancer growth. Treating mCSPC involves systemic therapy with ADT aimed at further reducing the tumor burden, enhancing disease control, and prolonging survival. In 2015, the CHAARTED and STAMPEDE arm C trials demonstrated an OS benefit of combining docetaxel with ADT over ADT alone, establishing this doublet therapy as the new standard of care for mCSPC [33]. Other therapeutic options, such as metformin combined with ADT, also showed effective management of mCSPC in recent clinical trials [34], remaining an area of ongoing investigation.

Recent RCTs have proven that combining ARSIs with ADT improves OS and progression-free survival (PFS) [10,11,13,15,16,20,22,24,26,27]. In the overall population of our network meta-analysis (NMA), ARSI + ADT doublet therapies, including abiraterone acetate, apalutamide, enzalutamide, and rezvilutamide, have better survival outcomes compared to ADT alone, with the exception of orteronel. Orteronel, also known as TAK-700, is a new CYP17 inhibitor that exhibits better specificity in targeting CYP17, 20-lyase than abiraterone acetate, inhibiting androgen synthesis [27]. However, according to our P-score ranking, orteronel has the lowest probability of being the best treatment regarding OS.

In mCSPC patients with a Gleason score of ≥8 or <8, the survival outcomes vary significantly. In the Gleason score ≥8 subgroup, all ARSI combination therapies demonstrated better OS compared to ADT alone, with rezvilutamide showing the highest probability of being the best treatment (P-score 0.88). In the CHART trial [26], Gu et al. indicated that rezvilutamide (formerly SHR3680), a new androgen receptor inhibitor featuring low blood–brain barrier penetration, showed improvement of survival outcome, especially in high-volume mCSPC. Conversely, in mCSPC patients with a Gleason score of <8, not all ARSI combination therapies significantly improved OS compared to ADT alone. Moreover, ADT combined with docetaxel significantly enhanced OS (HR 0.75, 95% CI 0.59–0.95), suggesting that docetaxel + ADT may still play a crucial role in the treatment of low-grade mCSPC. These findings might have implications for clinical treatment strategies.

In comparison, Fizazi et al. [29] and Smith et al. [28] demonstrated that triplet therapies combining ADT + docetaxel with either abiraterone acetate or darolutamide are effective. In our NMA, darolutamide + docetaxel + ADT improved OS compared to ADT alone. In terms of P-score, this combination treatment had the highest, second highest, and highest probability of being the best treatment in the overall population, Gleason score ≥8 subgroup, and Gleason score < 8 subgroup, respectively. Similarly, abiraterone acetate + docetaxel + ADT improved OS in the overall population but was not included in the Gleason score ≥8 or <8 subgroups due to the lack of Gleason score data. Future research should focus on comparing the side effects of triplet and doublet combination therapies to evaluate their efficacy and adverse effects for clinical treatment guidance.

The Gleason grading system was first developed in the 1960s, with significant updates by Gleason and Mellinger in 1974 [35] and by the International Society of Urological Pathology [36] in 2005. Once the Gleason score is 8 or above, the tumor prognosis worsens dramatically [37]. A Gleason score of 8 or higher predicts poor outcomes in patients undergoing castration [38,39,40]. The association between Gleason score and treatment efficacy has rarely been addressed. Understanding the differences in survival outcomes among patients with a varying Gleason score treated with different types of ARSI can aid in clinical treatment selection. Although there is a previous NMA that explored subgroup analysis of the Gleason score, it only compared doublet therapies [41]. In contrast, our NMA expands the treatment combinations and compares both triplet and doublet therapies, aligning more closely with current guidelines.

This study has several limitations to consider. First, the proportion of high or low disease volume varied across different studies. The CHART trials [26] included all patients with high-volume mCSPC. These differences can lead to variations in treatment outcomes and may introduce different results for efficacy ranking. Second, heterogeneous patient characteristics were found in the included trials. In the PEACE-1 trial [29], it is challenging to clarify the effect of radiotherapy. Moreover, the choice of standard nonsteroidal anti-androgen (NSAA) drug (bicalutamide, nilutamide, or flutamide) may differ in the control arms of the CHART [26], ENZAMET [24,25], and SWOG-1216 [27] trials. Despite the inconsistency in follow-up durations among the trials, most studies had relatively long follow-up periods, except for the CHART trials [26], which only accomplished an interim analysis (median follow-up: 21.2 months). Third, the safety profile of NSAA is not considered in this study. Serious adverse events may influence therapeutic preference and need further evaluation. Fourth, regarding PFS, although radiographic progression-free survival (rPFS) and clinical PFS (cPFS) are recognized as valid surrogates for OS in mCSPC, we did not differentiate between cPFS and rPFS, which could potentially bias the results. Fifth, although we compared the outcomes within the high and low Gleason score groups, we did not perform a direct comparison between the two groups. Future studies could focus on analyzing the differences in drug efficacy between the high and low Gleason score groups to further inform clinical decision making and enhance the validity of treatment selection. Finally, our NMA is based on indirect comparisons to allow for the comparison of different studies; however, the findings should be interpreted with caution and not as a replacement for direct evidence from RCTs.

In conclusion, this is the first study to compare the performance of standard care with different ARSIs in combination with ADT and/or docetaxel for treating mCSPC in patients with a Gleason score of ≥8 or <8. In the overall population, most ADT-based systemic therapies combined with ARSIs and/or docetaxel significantly improved OS compared to ADT alone, with the exception of orteronel + ADT. Specifically, in the Gleason score ≥8 subgroup, all therapies with ARSI + ADT or ARSI + docetaxel + ADT improved OS compared to ADT alone. However, in the Gleason score <8 subgroup, only ADT combined with darolutamide + docetaxel, apalutamide, or docetaxel showed an improvement in OS. These findings suggest that while an ARSI combined with ADT and/or docetaxel is an effective treatment option for mCSPC patients with a Gleason score of ≥8, further investigation is needed to determine its efficacy in patients with a Gleason score of <8.

## Figures and Tables

**Table 1:** Study characteristics of the 12 studies.

Trial	Author	Overall Population (E vs. C)	Experimental Arm	Control Arm	Median Age, Years (E vs. C)	Gleason Score < 8, No. (%) (E vs. C)	Gleason Score ≥ 8, No. (%) (E vs. C)	Median Follow-Up, Months	Primary Endpoint
ARASENS	Smith et al. [28]	651/654	Darolutamide + DOC + ADT	DOC + ADT	67/67	122 (18.7)/118 (18.0)	505 (77.6)/516 (78.9)	43.7	OS
ARCHES	Armstrong et al. [22,23]	574/576	Enzalutamide + ADT	ADT	70/70	171 (29.8)/187 (32.5)	386 (67.2)/373 (64.8)	44.6	OS, rPFS
CHAARTED	Sweeney et al. [9,11]	397/393	DOC + ADT	ADT	64/63	117 (29.5)/104 (26.5)	241 (60.7)/243 (61.8)	53.7	OS, cPFS
CHART	Gu et al. [26]	326/328	Rezvilutamide + ADT	Bicalutamide + ADT	69/69	47 (14)/64 (20)	276 (85)/257 (78)	29.3	OS, rPFS
ENZAMET	Davis et al. [24,25]	563/562	Enzalutamide + ADT	NSAA + ADT	69/69	152 (27)/163 (29)	335 (60)/321 (57)	68.0	OS, cPFS
GETUG-AFU15	Gravis et al. [13,14]	192/193	DOC + ADT	ADT	63/64	84 (45)/78 (41)	103 (55)/113 (59)	83.9	OS, rPFS
LATITUDE	Fizazi et al. [15,17]	597/602	Abiraterone + ADT	ADT	67.3/66.8	13 (3)/16 (3)	584 (98)/586 (97)	51.8	OS, rPFS
PEACE-1	Fizazi et al. [29]	355/355	Abiraterone + DOC + ADT + RT(+/−)	DOC + ADT + RT(+/−)	66/66	79 (23)/71 (20)	270 (77)/276 (80)	52.8	OS, rPFS
STAMPEDE Arm C	Clarke et al. [10]	362/724	DOC + ADT	ADT	65/65	65 (18)/158 (22)	253 (70)/480 (66)	78.2	OS, PFS
STAMPEDE Arm G	James et al. [19]	960/957	Abiraterone + ADT	ADT	67/67	221 (23)/223 (23)	715 (74)/721 (75)	73.0	OS, PFS
SWOG-1216	Agarwal et al. [27]	638/641	Orteronel + ADT	Bicalutamide + ADT	67.6/68.1	211 (33)/207 (33)	372 (58)/382 (60)	58.8	OS, PFS
TITAN	Chi et al. [20,21]	525/527	Apalutamide + ADT	ADT	69/68	174 (33.1)/169 (32.1)	351 (66.9)/358 (67.9)	44.0	OS, rPFS

ADT, androgen deprivation therapy; C, control arm; cPFS, clinical progression-free survival; DOC, docetaxel; E, experimental arm; NSAA, nonsteroidal anti-androgen (bicalutamide, nilutamide, or flutamide); OS, overall survival; PFS, progression-free survival; rPFS, radiographic progression-free survival; RT, radiotherapy.

## Data Availability

The data that support the findings of this study are available from the corresponding author upon reasonable request.

## Supplementary Materials

The following supporting information can be downloaded at: https://www.mdpi.com/article/10.3390/jcm14041326/s1, Table S1: Full search strategy in (1) Embase, (2) Web of Science, and (3) PubMed; Figure S1: Network plot of overall survival in metastatic castration-sensitive prostate cancer; Figure S2: Network plot of progression-free survival in metastatic castration-sensitive prostate cancer; Figure S3: Network plot of overall survival in patients with a Gleason score of ≥8; Figure S4: Network plot of overall survival in patients with a Gleason score of <8; Figure S5: Forest plot showing overall survival in metastatic castration-sensitive prostate cancer; Figure S6: Forest plot showing progression-free survival in metastatic castration-sensitive prostate cancer; Figure S7: Forest plot showing overall survival in patients with a Gleason score of ≥8; Figure S8: Forest plot showing overall survival in patients with a Gleason score of <8; Figure S9: Forest plot of hazard ratios for progression-free survival across different therapeutic regimens; Figure S10: Funnel plot for overall survival of the overall population; Figure S11: Funnel plot for progression-free survival of the overall population; Figure S12: Funnel plot for overall survival of patients with a Gleason score of ≥8; Figure S13: Funnel plot for overall survival of patients with a Gleason score of <8.
