# Supplementary material for: The Predictive Role of the Gleason Score in Determining Prognosis to Systematic Treatment in Metastatic Castration-Sensitive Prostate Cancer: A Systematic Review and Network Meta-Analysis
Source: J Clin Med. 2025 Feb 17;14(4):1326. doi: 10.3390/jcm14041326 (PMC11857080; doi:10.3390/jcm14041326)
Supplement: Supplementary file 1 [file jcm-14-01326-s001.zip › jcm-3437841-supplementary.pdf]

Supplementary Table S1. Full search strategy in (1) Embase, (2) Web of Science, and (3) PubMed.

1. **Search strategy in Embase**
("prostate cancer"/exp OR "prostate carcinoma"/exp OR "prostatic cancer"/exp OR "prostatic carcinoma"/exp) AND ("metastatic"/exp OR 'M1"/exp OR "advanced"/exp) AND ("enzalutamide"/exp OR "darolutamide"/exp OR "apalutamide"/exp OR 'abiraterone acetate"/exp OR "rezvilutamide"/exp OR "orteronel"/exp OR "docetaxel"/exp OR "anti-androgens"/exp OR "antiandrogens"/exp OR "hormonal therapy"/exp) AND "randomized controlled trial"/exp
2. **Search strategy in Web of Science**
TS=((Prostate cancer OR prostate carcinoma OR prostatic cancer OR prostatic carcinoma) AND (metastatic OR M1 OR Advanced) AND (enzalutamide OR darolutamide OR apalutamide OR abiraterone acetate OR rezvilutamide OR orteronel OR docetaxel OR anti-androgens OR antiandrogens OR hormonal therapy) AND randomized)
3. **Search strategy in PubMed**
((Prostate cancer[MeSH Terms]) OR (prostate carcinoma) OR (prostatic cancer) OR (prostatic carcinoma)) AND ((metastatic) OR (M1) OR (Advanced)) AND ((enzalutamide) OR (darolutamide) OR (apalutamide) OR (abiraterone acetate) OR (rezvilutamide) OR (orteronel) OR (docetaxel) OR (anti-androgens) OR (antiandrogens) OR (hormonal therapy)) AND (randomized controlled trial(Publication Type))

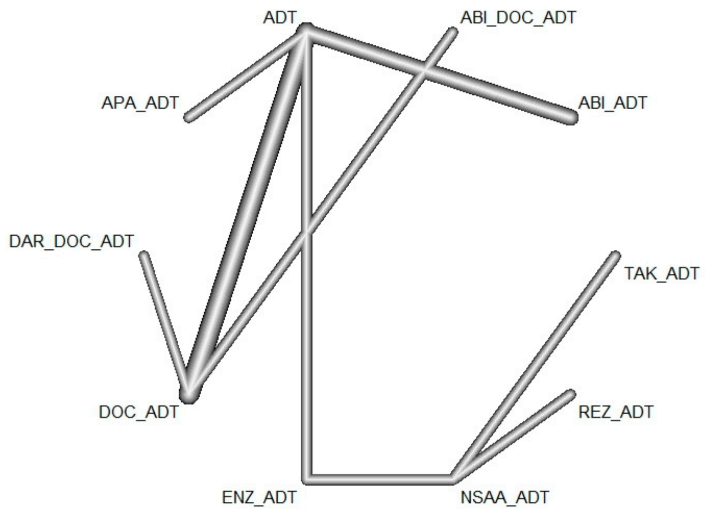

Supplementary Figure S1. Network plot of overall survival in metastatic castration-sensitive prostate cancer. ABI, abiraterone acetate; ADT, androgen deprivation therapy; APA, apalutamide; DAR, darolutamide; DOC, docetaxel; ENZ, enzalutamide; NSAA, standard nonsteroidal antiandrogen (bicalutamide, nilutamide, or flutamide); REZ, rezvilutamide; TAK, orteronel.

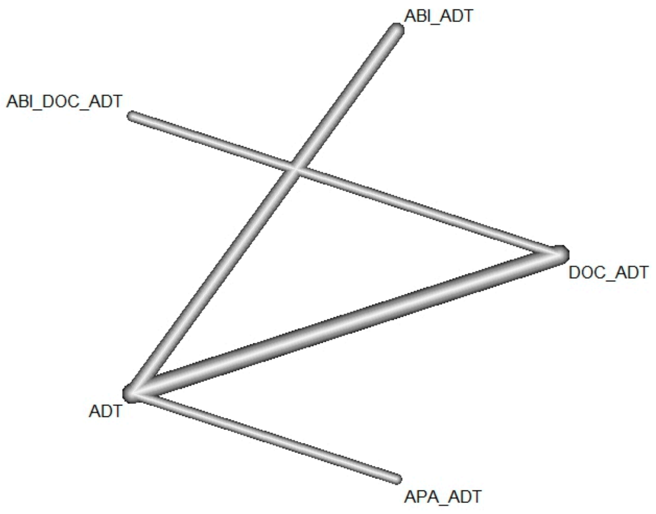

Supplementary Figure S2. Network plot of progression-free survival in metastatic castration-sensitive prostate cancer. ABI, abiraterone acetate; ADT, androgen deprivation therapy; APA, apalutamide; DOC, docetaxel.

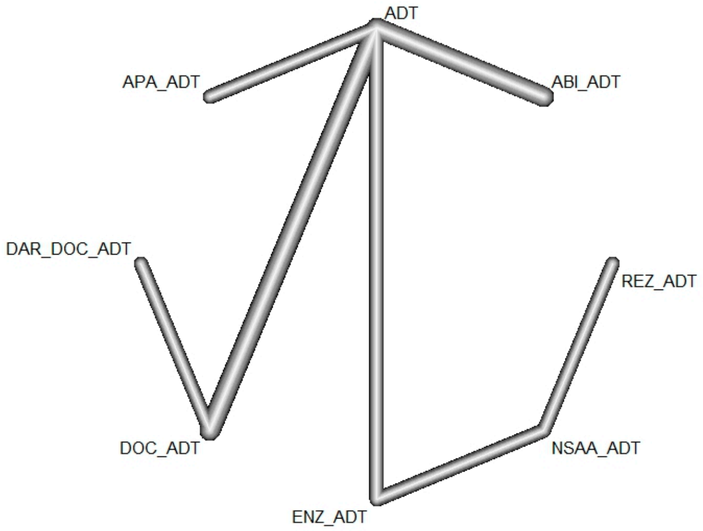

Supplementary Figure S3. Network plot of overall survival in patients with a Gleason score of ≥8. ABI, abiraterone acetate; ADT, androgen deprivation therapy; APA, apalutamide; DAR, darolutamide; DOC, docetaxel; ENZ, enzalutamide; NSAA, standard nonsteroidal antiandrogen (bicalutamide, nilutamide, or flutamide); REZ, rezvilutamide.

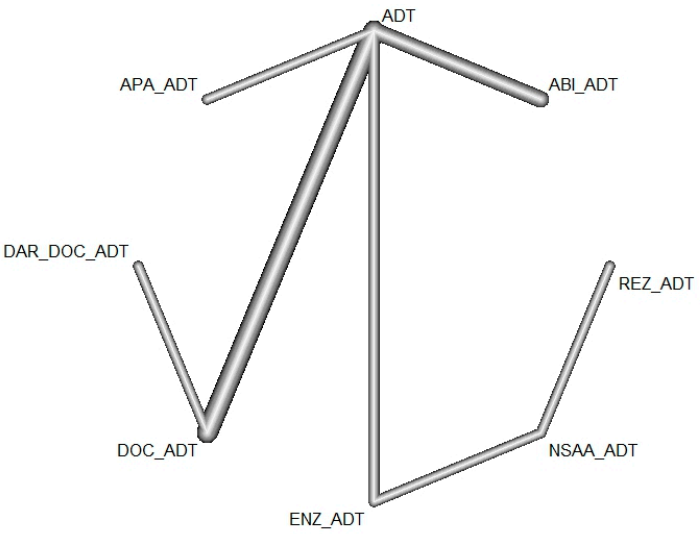

Supplementary Figure S4. Network plot of overall survival in patients with a Gleason score of <8. ABI, abiraterone acetate; ADT, androgen deprivation therapy; APA, apalutamide; DAR, darolutamide; DOC, docetaxel; ENZ, enzalutamide; NSAA, standard nonsteroidal antiandrogen (bicalutamide, nilutamide, or flutamide); REZ, rezvilutamide.

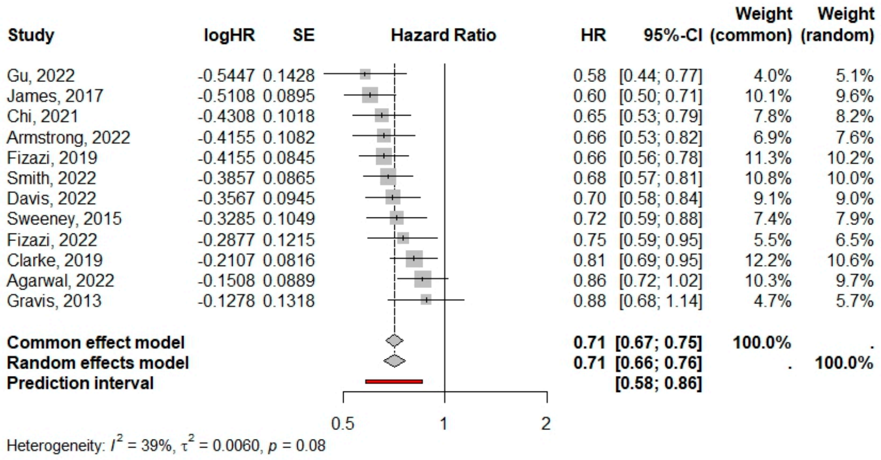

Supplementary Figure S5. Forest plot showing overall survival in metastatic castration-sensitive prostate cancer [9–11,13–15,17,19–29].

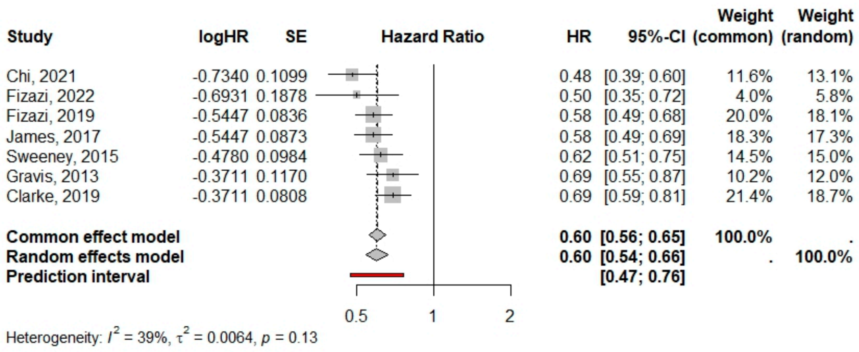

Supplementary Figure S6. Forest plot showing progression-free survival in metastatic castration-sensitive prostate cancer [9–11,13–15,17,19–21,29].

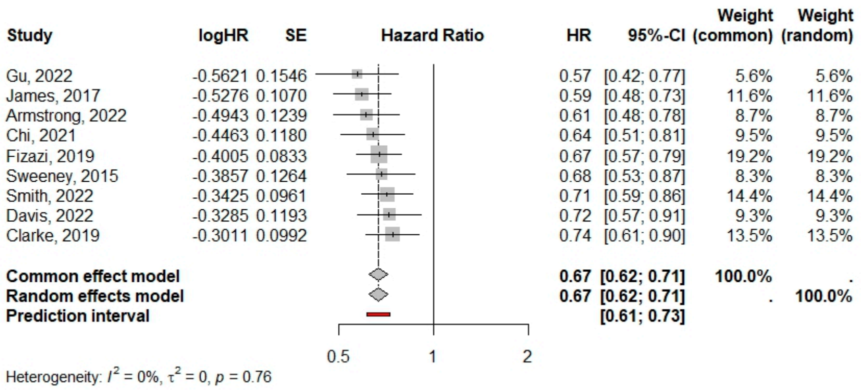

Supplementary Figure S7. Forest plot showing overall survival in patients with a Gleason score of ≥8 [9–11,15,17,19–26,28].

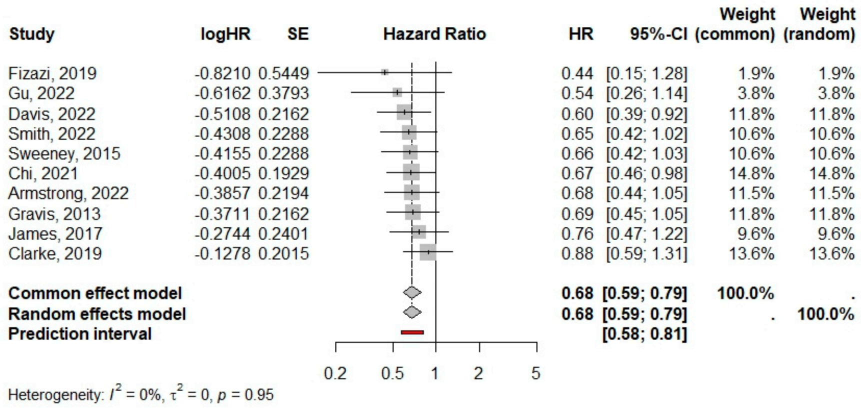

Supplementary Figure S8. Forest plot showing overall survival in patients with a Gleason score of <8 [9–11,13–15,17,19–26,28].

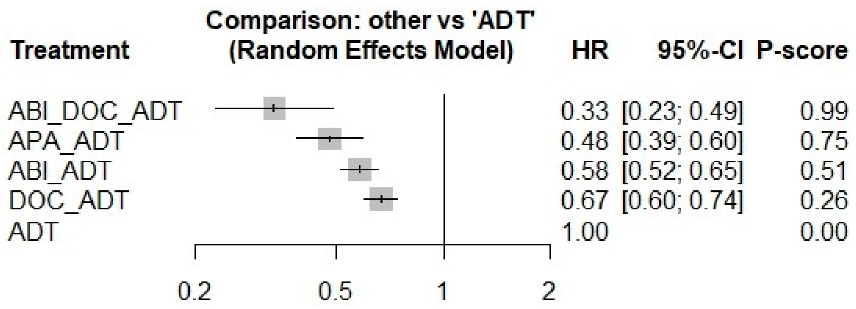

Supplementary Figure S9. Forest plot of hazard ratios for progression-free survival across different therapeutic regimens. ABI, abiraterone acetate; ADT, androgen deprivation therapy; APA, apalutamide; DOC, docetaxel.

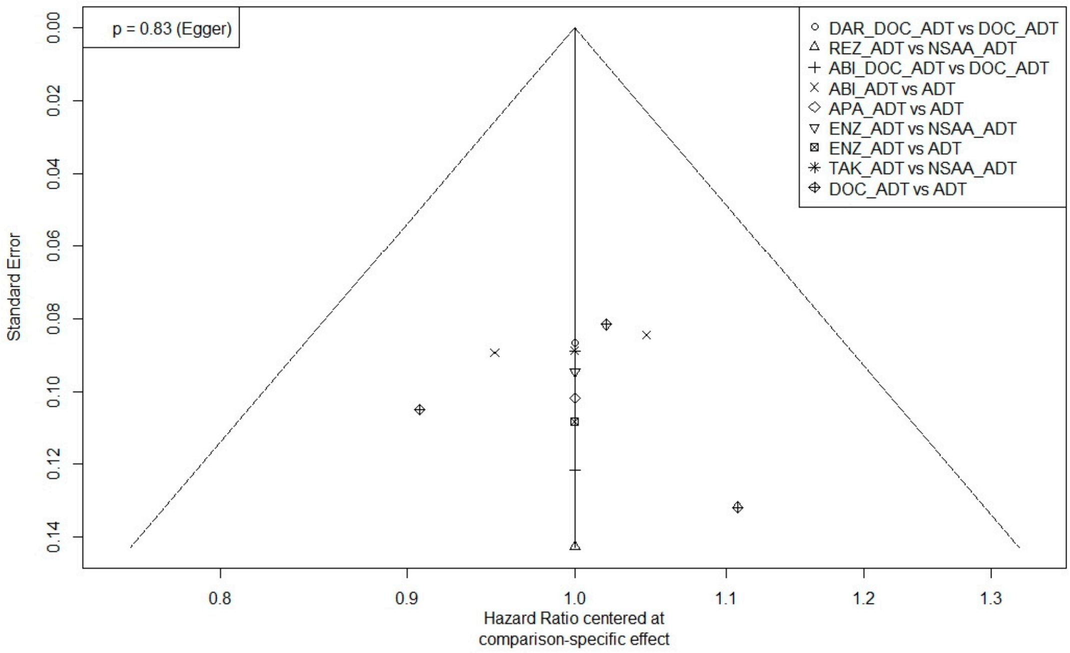

Supplementary Figure S10. Funnel plot for overall survival of the overall population. ABI, abiraterone acetate; ADT, androgen deprivation therapy; APA, apalutamide; DAR, darolutamide; DOC, docetaxel; ENZ, enzalutamide; NSAA, standard nonsteroidal antiandrogen (bicalutamide, nilutamide, or flutamide); REZ, rezvilutamide; TAK, orteronel.

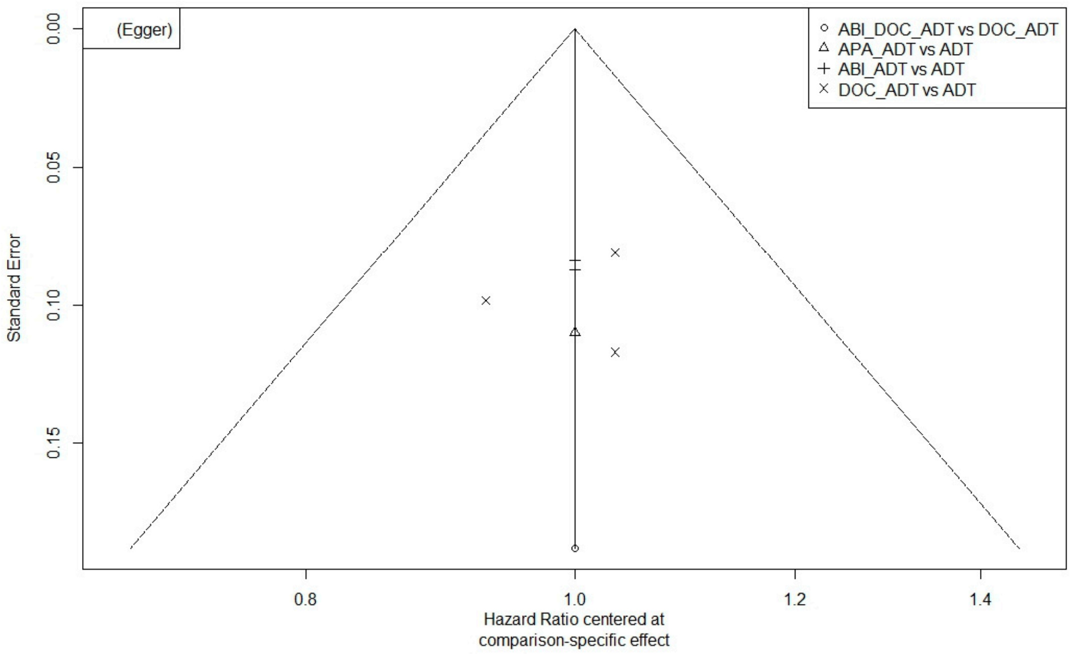

Supplementary Figure S11. Funnel plot for progression-free survival of the overall population. ABI, abiraterone acetate; ADT, androgen deprivation therapy; APA, apalutamide; DOC, docetaxel.

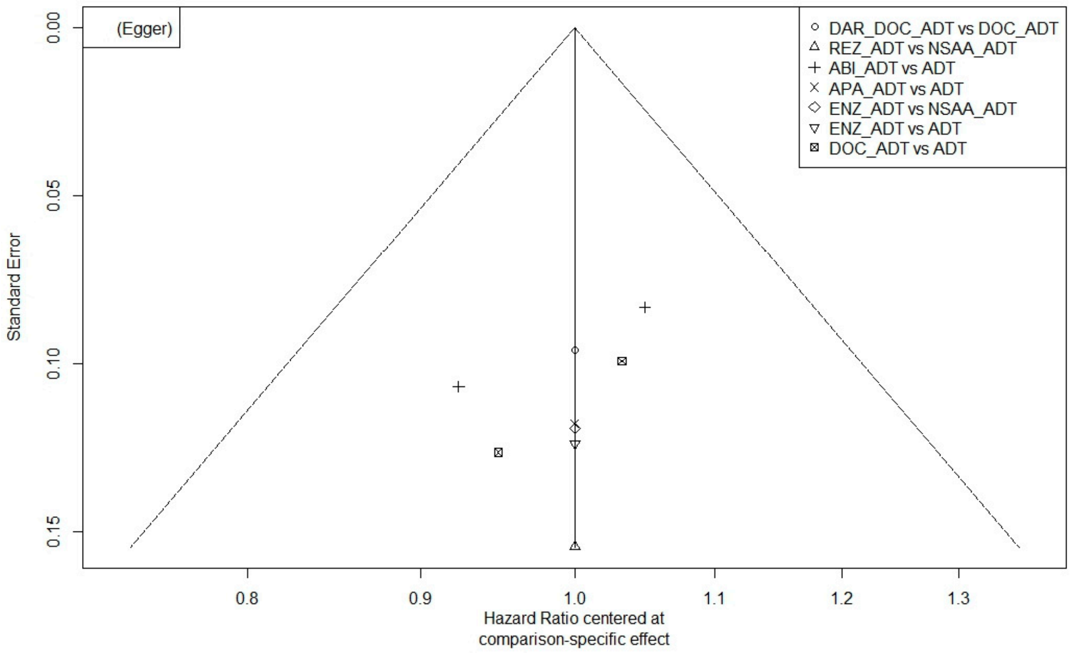

Supplementary Figure S12. Funnel plot for overall survival of patients with a Gleason score of ≥8. ABI, abiraterone acetate; ADT, androgen deprivation therapy; APA, apalutamide; DAR, darolutamide; DOC, docetaxel; ENZ, enzalutamide; NSAA, standard nonsteroidal antiandrogen (bicalutamide, nilutamide, or flutamide); REZ, rezvilutamide.

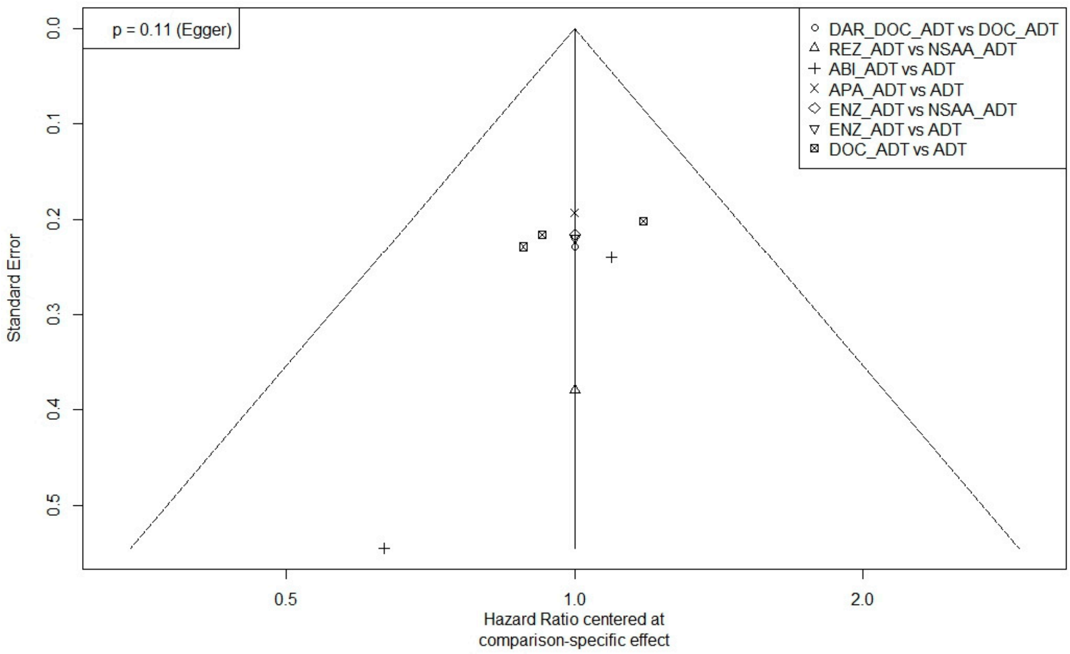

Supplementary Figure S13. Funnel plot for overall survival of patients with a Gleason score of <8. ABI, abiraterone acetate; ADT, androgen deprivation therapy; APA, apalutamide; DAR, darolutamide; DOC, docetaxel; ENZ, enzalutamide; NSAA, standard nonsteroidal antiandrogen (bicalutamide, nilutamide, or flutamide); REZ, rezvilutamide.
